# Effects of Tai Chi on working memory in older adults: evidence from combined fNIRS and ERP

**DOI:** 10.3389/fnagi.2023.1206891

**Published:** 2023-06-29

**Authors:** Chen Wang, Yuanfu Dai, Yuan Yang, Xiaoxia Yuan, Mengjie Zhang, Jia Zeng, Xiaoke Zhong, Jiao Meng, Changhao Jiang

**Affiliations:** ^1^The Center of Neuroscience and Sports, Capital University of Physical Education and Sports, Beijing, China; ^2^College of Physical Education and Sports, Beijing Normal University, Beijing, China; ^3^School of Physical Education and Sport Science, Fujian Normal University, Fuzhou, China; ^4^School of Kinesiology and Health, Capital University of Physical Education and Sports, Beijing, China

**Keywords:** Tai Chi, older adults, working memory, ERP, fNIRS

## Abstract

**Objective:**

The study aimed to investigate the effects of a 12-week Tai Chi exercise intervention on working memory in older adults using ERP-fNIRS.

**Method:**

Fifty older adults were randomly assigned to either an experimental group receiving a 12-week Tai Chi exercise intervention or a control group receiving regular daily activities. Working memory was assessed using the n-back task before and after the intervention, and spatial and temporal components of neural function underlying the n-back task were measured using ERP-fNIRS.

**Results:**

The experimental group demonstrated significant improvements in reaction time and accuracy on the 2-back task and showed higher activation levels in the R-DLPFC. Additionally, the Tai Chi group displayed significant increases in P3 amplitude in the overall n-back task.

**Conclusion:**

These findings suggest that Tai Chi interventions can enhance working memory in older adults, as evidenced by increasing neural activity and improving HbO in the R-DLPFC during the 2-back task.

## 1. Introduction

Aging is associated with degenerative changes in brain structure and function, which can result in a decline in working memory function (Oberauer and Lewandowsky, 2008). Cognitive decline can increase the risk of cognitive impairment, dementia, and other related diseases, which can have a negative impact on the quality of life in older adults. Working memory is a memory system that temporarily stores and manipulates language and image information essential for complex cognitive tasks, including language comprehension, learning, and reasoning (Baddeley, 2012). Studies have shown that working memory is plastic, meaning that it can be modified and improved through training and practice, and physical exercise interventions may be more effective in enhancing overall cognitive ability and improving memory in older adults than cognitive interventions (Diamond and Lee, 2011; Langlois et al., 2013; Tarumi et al., 2020).

Some researchers have investigated the use of mind-body exercises to delay the decline of cognitive function in older adults. Mind-body exercise involves physical activity that focuses on improving balance and flexibility while also controlling breathing and directing attention, such as the Eight Brocades or Tai Chi (Law et al., 2020). A meta-analysis of interventions using mind-body exercises in older adults found that including exercises such as Tai Chi and square dance exercise were beneficial in improving working memory in both cognitively intact and impaired older adults (Wu et al., 2019). Tai Chi, with its gentle and easy-to-learn movements, is one of the traditional Chinese sports and has become widely accepted (Wu et al., 2013). Research on Tai Chi has been increasing over the years, and our recent study divided older adults into Tai Chi experts and novices based on their length of practice found that the expert group outperformed the novice group in n-back tasks, indicating a positive impact of long-term Tai Chi practice on working memory in older adults (Yang et al., 2022). Another study has found that Tai Chi intervention improves inhibition control in older adults and increases oxyhemoglobin (HbO) in conflict tasks (Yang et al., 2020). However, the impact of Tai Chi on working memory in older adults and the underlying neural mechanisms are still unclear.

Previous studies have mainly used event-related potentials (ERPs) to measure cortical activity in the brain during cognitive task performance. ERPs can detect the temporal components of working memory processing, with the P3 component being a marker of attention allocation during working memory updating (Neuhaus et al., 2010). Physical activity interventions have been found to enhance P3 amplitude in older adults during working memory tasks (Chang et al., 2013). However, spatial neural changes are difficult to measure using ERPs. To facilitate the observation of brain hemodynamic changes, we also employed functional near-infrared spectral (fNIRS), fNIRS is a non-invasive neuroimaging method that uses near-infrared light to monitor cerebral hemodynamic parameters continuously. It is widely used to monitor prefrontal cortex (PFC) activity during cognitive task performance (Leff et al., 2011; Yücel et al., 2017; Pinti et al., 2020). The use of ERP-fNIRS joint measurement allowed the identification of the spatial and temporal components of the neural functional basis of working memory processing.

Therefore, the aim of this study was to investigate the effects of a 12-week Tai Chi intervention using the n-back task on working memory in healthy older adults. Additionally, ERP-fNIRS combined measurements were utilized to assess differences in brain activity during task performance. The hypothesis was that older adults in the Tai Chi group would demonstrate better n-back performance and higher brain HbO concentration and P3 amplitude during the task compared to the control group after the 12-week intervention.

## 2. Materials and methods

### 2.1. Participants

Based on a G * Power 3.1 (Faul et al., 2007) estimation (*f* = 0.25, α = 0.05, 1−β = 0.80, two groups), we determined the minimum sample size of the current study to be 34 participants.

Fifty elderly participants from Xicheng District, Beijing were recruited using the following eligibility criteria: (1) age ≥ 65 years, without brain or neurological damage and in generally good health; (2) a Mini-Mental State Examination (MMSE) (Mitchell, 2009) score > 24; (3) right-handedness; (4) no experience in Tai Chi. Participants were randomly assigned to either a Tai Chi or control group.

Two participants in the control group were excluded from the analysis due to significant data artifacts caused by head movement (fNIRS), while one participant in the experimental group was excluded due to abnormally high impedance during the post-test (EEG). The final study included 47 participants. Demographic characteristics of both groups are shown in Table 1.

**TABLE 1:** Subjects’ demographic characteristics (M ± SD).

	Control group (*N* = 24)	TAI CHI group (*N* = 23)
Age (year)	66.33 ± 0.96	66.35 ± 0.98
Weight (kg)	66.13 ± 3.89	66.57 ± 4.69
Height (m)	1.65 ± 0.07	1.64 ± 0.06
MMSE	27.83 ± 1.65	27.91 ± 1.47

No significant difference in any characteristic was detected among groups (all *p* > 0.05).

### 2.2. Tai Chi intervention

The Tai Chi group participated in a 12-week intervention consisting of Tai Chi (Eight Forms Five Steps), which involves eight techniques and five steps of basic movements in Tai Chi practice. The techniques include Peng (ward-off), Lu (roll-back), Ji (press), An (push), Cai (pluck), Lie (split), Zhou (elbow strike), and Kao (shoulder strike), while the five steps include advance, retreat, look left, gaze right, and stand upright. The exercise intensity was maintained at a moderate level, with an average heart rate ranging from 60 to 69% of the maximum predicted heart rate, which is calculated by subtracting the participant’s age from 220. Each session lasted for 60 min, including a 5-min warm-up, a 50-min Tai Chi (Eight Forms Five Steps) practice, and a 5-min cool-down. A Tai Chi instructor with over 10 years of experience will teach Tai Chi, with a teaching assistant who has more than 5 years of Tai Chi experience. The intervention was conducted Monday, Wednesday, and Friday from 8:00 am to 9:00 am. Participants will only be allowed to participate in the prescribed Tai Chi exercise during the experimental period.

The control group was instructed to maintain their normal lifestyle and not to engage in any other physical activities.

### 2.3. Working memory assessment

**FIGURE 1:**
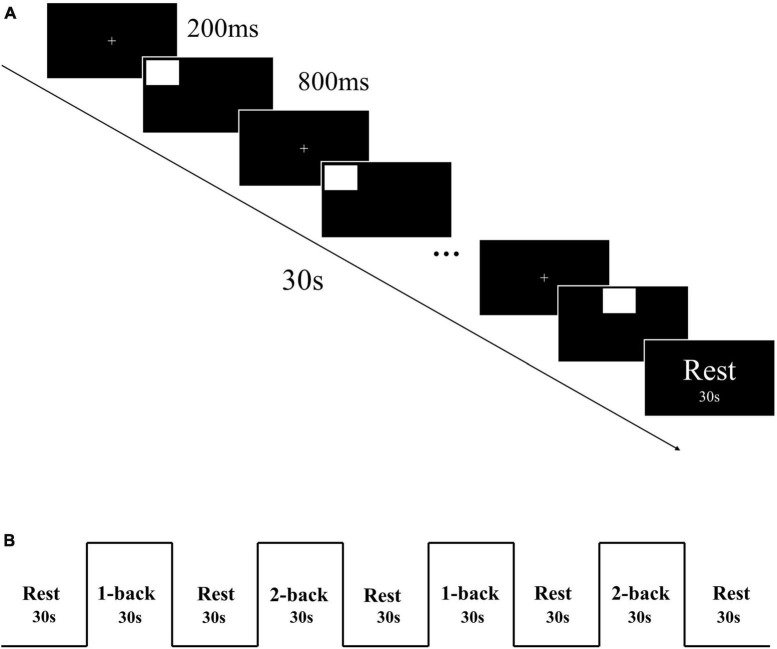
**(A)** Each block of n-back task. **(B)** n-back task block design.

The spatial n-back task, which included 1-back and 2-back tasks, was used to assess working memory. The task was programmed using E-prime 3.0 software. Initially, a “ + ” symbol was presented in the center of the screen for a duration of 200 ms, followed by the stimulus material for 800 ms. Participants were required to judge whether the current stimulus position matched the position of the stimulus presented n positions ago. An “F” key press indicated a match, and a “J” key press indicated a mismatch (Figure 1A). Participants had 800 ms to respond. The task was designed using a block design, with each task consisting of 2 blocks, each containing 30 trials. Each block lasted for 30 s, with a 30 s rest period in between (Figure 1B). Collected reaction time and accuracy of each participant.

### 2.4. fNIRS data acquisition and processing

**FIGURE 2:**
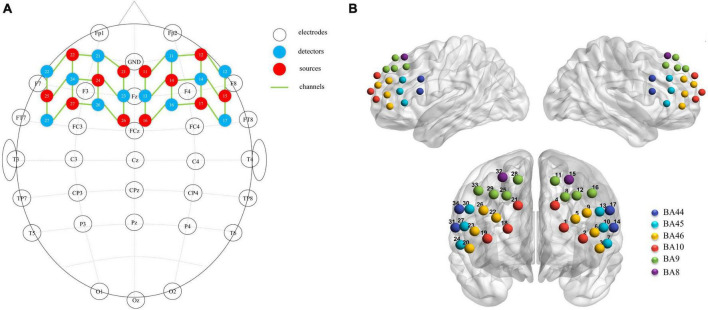
**(A)** fNIRS optodes (red = source, blue = detector) and EEG electrodes (white) mapped on the brain. **(B)** fNIRS channels mapped on the brain. One source and one detector formed a channel, and 34 channels were formed.

A near-infrared spectroscopy device (ETG-4000) from Hitachi Medical Corporation was used in this experiment. The device employs two wavelengths of near-infrared light, 695 and 830 nm, and samples the blood oxygen signal at a frequency of 10 Hz. Twelve emitting and 12 detecting optical fibers were inserted into a custom EEG-fNIRS cap based on the 10–20 system and positioned in the PFC (Figure 2A). Each emitting fiber was positioned 3 cm from its corresponding detecting fiber, forming a channel. A total of 34 channels were formed (Figure 2B). The fNIRS channel positions were registered with MNI space coordinates using a probability registration method, and the correspondences between the registered channels and Brodmann areas were determined (Rorden and Brett, 2000; Ye et al., 2009). Six Brodmann areas were covered, each of which contained specific channel positions as shown in Table 2. The regions of interest ROIs in this study corresponded to the following Brodmann areas: Frontopolar Area (FPA) – BA10; Dorsolateral prefrontal cortex (DLPFC) – BA46, BA9; Ventrolateral prefrontal cortex (VLPFC) – BA45, BA44; as BA8 only covered two channels (CH15, CH32), it was not studied in this article. The specific channels corresponding to each ROI were: R-FPA: CH18, CH19, CH21; L-FPA: CH1, CH2, CH4; R-DLPFC: CH25, CH28, CH29, CH33, CH22, CH20, CH23, CH26; L-DLPFC: CH8, CH11, CH12, CH16, CH5, CH3, CH6, CH9; R-VLPFC: CH31, CH34, CH24, CH27, CH30; L-VLPFC: CH14, CH17, CH7, CH10, CH13.

**TABLE 2:** Location for each channel.

Channels	MNI coordinates			Brodmann area	ROIs
	X	Y	Z		
CH18	22	71	15	10	R-FPA
CH19	38	64	7		
CH21	14	63	34		
CH25	23	53	42	9	R-DLPFC
CH28	14	42	55		
CH29	33	44	43		
CH33	45	31	46		
CH22	31	62	24	46	
CH20	52	49	−1		
CH23	48	52	13		
CH26	41	51	30		
CH31	63	16	16	44	R-VLPFC
CH34	60	16	31		
CH24	59	32	2	45	
CH27	56	37	17		
CH30	52	35	31		
CH1	−22	69	16	10	L-FPA
CH2	−37	63	7		
CH4	−15	62	34		
CH8	−23	52	41	9	L-DLPFC
CH11	−15	42	54		
CH12	−32	43	42		
CH16	−44	30	44		
CH5	−31	61	23	46	
CH3	−50	48	−1		
CH6	−46	51	12		
CH9	−40	49	28		
CH14	−61	15	16	44	L-VLPFC
CH17	−58	14	30		
CH7	−56	32	3	45	
CH10	−53	37	16		
CH13	−50	34	29		
CH15	−26	32	55	8	–
CH32	26	33	57		

L, left; R, right; MNI, Montreal Neurological Institute.

The fNIRS data were processed using NIRS_SPM (MGHMartinos Center for Biomedical Imaging, Boston, MA, USA) (Ye et al., 2009) based on Matlab (Mathworks Natick, Boston, MA, USA). Our study employed the Wavelet_MDL algorithm to detrend the fNIRS data. Specifically, the algorithm decomposed the NIRS measurements into globally trending, hemodynamic, and unrelated noise components at different scales using wavelet transforms. To prevent overfitting or underfitting of the global trend estimation, the minimum description length principle was applied. In addition, the Pre-coloring method (Worsley and Friston, 1995) was chosen, and its kernel function can be implemented using either a Gaussian filter or a hemodynamic response function (HRF) low-pass filter. The HRF filter used in this study is the preferred filter for fNIRS data because the transfer function of HRF is modeled on neuronal signals’ frequency (Ye et al., 2009), which can remove random noise generated by the instrument as well as physiological noise caused by heart rate, respiration, and so on. In the second step, the reference waves for the HbO changes for each experimental condition were set based on the general linear model (GLM), and the data were evaluated accordingly. In the third step, the changes in HbO for each channel were calculated using NIRS_SPM software and represented as beta values. The beta values for the region of interest were computed as the mean of the channels within each ROI.

### 2.5. Electroencephalographic data acquisition and processing

Electroencephalographic (EEG) data were collected using the NeuroScan Synamps2 EEG recording and analysis system (Compumedics NeuroScan Inc., Charlotte, NC, USA) in accordance with the international standard 10–20 system, using a 32-electrode cap (see Figure 2A). The left mastoid electrode served as a reference, and the ground electrode was placed in front of FZ. Two electrodes above and below the left eye recorded vertical eye movements, and two electrodes placed 1.5 cm lateral to each eye recorded horizontal eye movements. Data were acquired with reference to CPz, and electrode impedances were maintained below 5 kΩ.

Offline data analysis was performed using the EEGLAB toolbox (Delorme and Makeig, 2004) in MATLAB. The data were manually screened for bad segments, and a bandpass filter was applied in the range of 0.1–30 Hz to remove 50 Hz power line noise. The analysis epoch was set to 1,000 ms, including 200 ms before and 800 ms after stimulus onset, with a baseline of 200 ms before stimulus onset. Independent component analysis (ICA) was used to remove eyeblink and cardiac artifacts, and trials contaminated by motion artifacts were excluded using a ± 100 μV threshold. The EEG signals from each participant under the task condition were averaged separately after referencing to bilateral mastoids (A1 and A2).

Based on observations of the averaged waveform and previous research (Chang et al., 2013; Yerlikaya et al., 2018), the P3 component of the ERP was defined as the maximum positive peak appearing between 250 and 550 ms after stimulus onset, with the amplitude of P3 generally being larger along the midline electrodes. Therefore, only Fz, Cz, and Pz electrodes were included in the statistical analysis.

### 2.6. Statistical analysis

Normal distribution was assumed for all data. A repeated-measures analysis of variance (ANOVA) was performed using IBM SPSS Statistics (SPSS Inc., Chicago, IL, USA) to analyze the behavioral data (reaction time [RT] and accuracy [ACC]), functional near-infrared spectroscopy (fNIRS) beta values, and event-related potential (ERP) P3 amplitude. The design included three factors: test time (pre-test, post-test), group (control, Tai Chi), and n-back level (1-back, 2-back). After the S-W normality test, it was determined that the data followed a normal distribution. To correct for degrees of freedom, the Greenhouse-Geisser method was employed, and the Bonferroni method was utilized for *post hoc* multiple comparison correction. An alpha level of 0.05 was used for all statistical tests.

## 3. Results

### 3.1. Effects of Tai Chi on working memory performance

**FIGURE 3:**
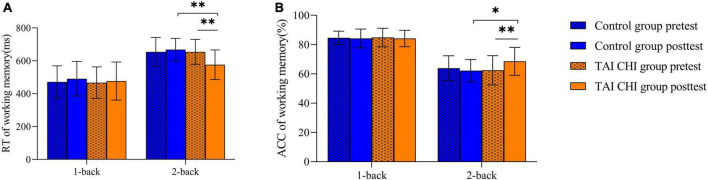
RTs **(A)** and ACCs **(B)** on the n-back task. *p < 0.05, ^**^p < 0.01, error bar means SD.

A repeated-measures analysis of variance (ANOVA) was performed on reaction time, which showed a significant main effect of task condition [*F*_(1, 45)_ = 367.285, *P* < 0.001, η*_*p*_*^2^ = 0.891]. The time × condition × group was significant [*F*_(1, 45)_ = 7.234, *P* = 0.010, η*_*p*_*^2^ = 0.138]. Further simple effect analyses indicated that in the 2-back task, the Tai Chi group exhibited significantly lower reaction time in the post-test compared to the pre-test [*F*_(1, 45)_ = 33.196, *P* < 0.001, η*_*p*_*^2^ = 0.425], and significantly lower reaction time in the post-test 2-back task compared to the control group [*F*_(1, 45)_ = 14.230, *P* < 0.001, η*_*p*_*^2^ = 0.240] (Figure 3A).

Similarly, a repeated-measures ANOVA was performed on accuracy, which showed a significant main effect of task condition [*F*_(1, 45)_ = 183.891, *P* < 0.001, η*_*p*_*^2^ = 0.803]. The time × condition × group interaction was significant [*F*_(1, 45)_ = 11.844, *P* = 0.001, η*_*p*_*^2^ = 0.208]. Further simple effect analyses showed that in the 2-back task, the Tai Chi group had significantly higher accuracy in the post-test compared to the pre-test [*F*_(1, 45)_ = 23.239, *P* < 0.001, η*_*p*_*^2^ = 0.341], and significantly higher accuracy in the post-test 2-back task compared to the control group [*F*_(1, 45)_ = 6.367, *P* = 0.015, η*_*p*_*^2^ = 0.124] (Figure 3B).

### 3.2. Effects of Tai Chi on fNIRS data

**FIGURE 4:**
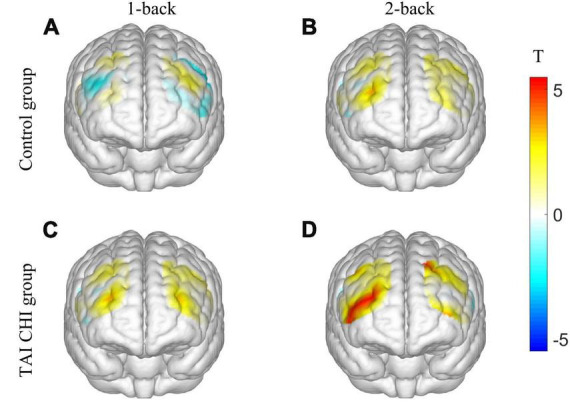
3D activation map. T-test results of control group before and after 1-back **(A)**. T-test results of control group before and after 2-back **(B)**. T-test results of TAI CHI group before and after 1-back **(C)**. T-test results of TAI CHI group before and after 2-back **(D)**.

Two participants from the control group were excluded from the analysis due to significant data artifacts caused by head movements. A repeated-measures analysis of variance (ANOVA) was conducted on the β values of the R-DLPFC. The ANOVA showed a significant main effect of time [*F*_(1, 45)_ = 4.714, *P* = 0.035, η*_*p*_*^2^ = 0.095] and task condition [*F*_(1, 45)_ = 24.120, *P* < 0.001, η*_*p*_*^2^ = 0.349]. Moreover, there was a significant interaction effect of time, task condition, and group [*F*_(1, 45)_ = 4.986, *P* = 0.031, η*_*p*_*^2^ = 0.100]. Further simple effects analysis revealed that the experimental group had significantly higher β values in the post-test compared to the pre-test in the 2-back task [*F*_(1, 45)_ = 5.770, *P* = 0.020, η*_*p*_*^2^ = 0.114], and significantly higher β in the post-test 2-back task compared to the control group [*F*_(1, 45)_ = 17.017, *P* < 0.001, η*_*p*_*^2^ = 0.274]. However, no significant differences were found in the other ROIs. Pairwise t-tests were conducted on the β values of each channel pre-test and post-test, and a 3D activation map was generated based on the results (Figure 4).

### 3.3. Effects of Tai Chi on ERP data

A repeated-measures ANOVA was conducted on the amplitudes of the ERPs at Fz. The ANOVA revealed a significant main effect of time [*F*_(1, 45)_ = 4.763, *P* = 0.034, η*_*p*_*^2^ = 0.096] and a significant time × group interaction [*F*_(1, 45)_ = 12.908, *P* = 0.01, η*_*p*_*^2^ = 0.223]. Further simple effects analysis demonstrated that during both 1-back and 2-back, the Tai Chi group had significantly higher amplitudes in the post-test than in the pre-test [*F*_(1, 45)_ = 17.039, *P* < 0.001, η*_*p*_*^2^ = 0.275], while there was no significant difference in the control group.

A repeated-measures ANOVA was also performed on the ERP amplitudes at Cz. The analysis revealed a significant time × group interaction [*F*_(1, 45)_ = 6.964, *P* = 0.011, η*_*p*_*^2^ = 0.134]. Further simple effects analysis showed that during both 1-back and 2-back, the Tai Chi group had significantly higher amplitudes in the post-test than in the pre-test [*F*_(1, 45)_ = 9.158, *P* = 0.004, η*_*p*_*^2^ = 0.169], while there was no significant difference in the control group.

Another repeated-measures ANOVA was conducted on the ERP amplitudes at Pz, revealing a significant main effect of time [*F*_(1, 45)_ = 16.021, *P* < 0.001, η*_*p*_*^2^ = 0.263] and a significant time × group interaction [*F*_(1, 45)_ = 47.639, *P* < 0.001, η*_*p*_*^2^ = 0.514]. Further simple effects analysis showed that during both 1-back and 2-back, the Tai Chi group had significantly higher amplitudes in the post-test than in the pre-test [*F*_(1, 45)_ = 60.749, *P* < 0.001, η*_*p*_*^2^ = 0.574], while the control group had significantly lower amplitudes in the post-test than in the pre-test [*F*_(1, 45)_ = 4.116, *P* = 0.048, η*_*p*_*^2^ = 0.084].

**FIGURE 5:**
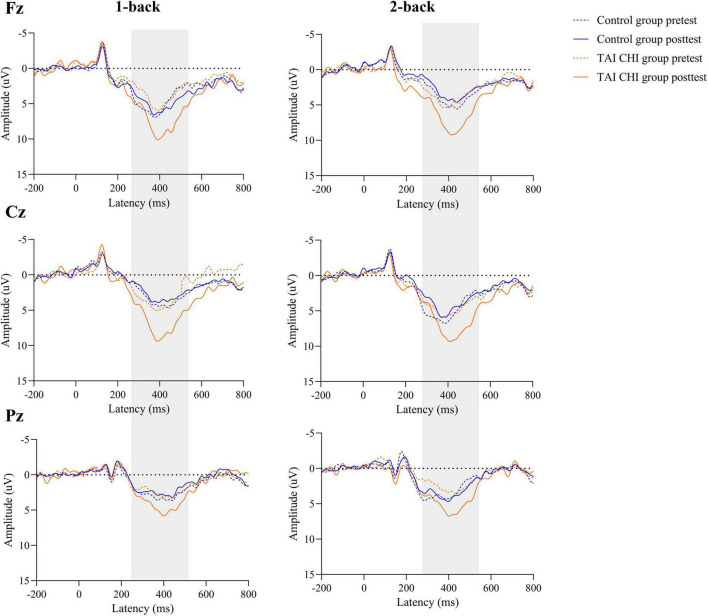
The grand average ERPs at the Fz, Cz, and Pz electrodes stratified by group.

The waveform plots for all ERPs are presented in Figure 5.

## 4. Discussion

This study aimed to investigate the impact of Tai Chi exercise on working memory in older adults, while obtaining fNIRS and ERP data during a working memory task to explore alterations in brain neurobiology. Our findings demonstrate that following 12-weeks of Tai Chi intervention, older adults exhibited improved performance on the working memory task, consistent with our hypothesis. Furthermore, we observed increased levels of neural activity, which supports the potential of Tai Chi exercise as an effective intervention for improving cognitive function in older adults.

Our study demonstrated that participants in the Tai Chi group exhibited significant improvements in 2-back scores following Tai Chi intervention. The 2-back task is a moderately complex working memory task, and our findings are in line with previous research (Lam et al., 2011; Sun et al., 2015). Additionally, Yu et al. (2022) found that older adults who participated in a 10-week Tai Chi intervention showed greater improvements in memory function compared to those in a fast-walking group. Furthermore, a meta-analysis by Wayne et al. (2014) that involved 20 studies and 2,553 older adults indicated that Tai Chi intervention was superior to other forms of exercise in enhancing cognitive function among older adults. Physical activity is known to have positive effects on cognition and brain function and can prevent the onset of neurodegenerative diseases (Kramer and Erickson, 2007). Tai Chi, being a mind-body exercise, combines cognitive activities such as action updating with physical movements that involve visual information processing. This integration engages both the cognitive and motor neural systems and has both cognitive training and aerobic exercise components (Yeh et al., 2008), which provides a possible explanation for the improvements in working memory observed among older adults who practice Tai Chi. Additionally, Steinborn and Huestegge (2016) found that rest during exercise benefits subjects’ ability to focus their attention. Tai Chi emphasizes the harmonious integration of softness and firmness, the combination of emptiness and substance, and rest is also an integral part of Tai Chi. Therefore, we believe that Tai Chi is an effective means for older adults to improve attention and memory abilities.

We utilized fNIRS to examine changes in activation of the R-DLPFC during a 2-back task following a Tai Chi intervention. Our findings showed that the β values in the R-DLPFC of the Tai Chi group significantly increased from pre- to post-test, indicating increased activation during the 2-back task. β values reflect the concentration of HbO hemoglobin in the ROI (Ye et al., 2009), and higher β values suggest increased HbO in the R-DLPFC of the Tai Chi group during the post-test. Previous research has shown that long-term high-intensity aerobic exercise positively affects the concentration levels of the hemodynamic response related to the L - DLPFC (Chen et al., 2020). The DLPFC is a crucial brain region for working memory, and decreased activity in this region is associated with poor performance on working memory tasks (Menon and D’Esposito, 2022). The R-DLPFC is particularly important for manipulating information in complex environments (Barbey et al., 2013), and has been suggested to be the dominant hemisphere for spatial working memory (McCarthy et al., 1996; Lundstrom et al., 2005). The current study employed a spatial n-back task, which explains why the DLPFC was located in the right hemisphere following the intervention. Exercise is an effective method for accelerating blood flow supply and enhancing local cerebral blood flow, and moderate-intensity exercise is beneficial for cerebral blood flow supply (Livingston et al., 2017).

In contrast to the behavioral and fNIRS data, the Tai Chi group showed significant increases in P3 amplitude at all electrodes during not only the 2-back but also the 1-back task, while the control group did not show a significant difference. This finding is consistent with previous research. Chang et al. (2013) divided elderly individuals into a high physical activity group and a low physical activity group and found that the high physical activity group had better performance and higher P3 amplitude during the n-back task. Lenartowicz et al. (2021) utilized EEG to measure brain electrical signals in 200 participants during a working memory task and found that P3 is one of the critical factors that predicts working memory ability. P3 reflects cognitive or attentional resource allocation, and its amplitude reflects the amount of cognitive resources allocated during the retrieval phase (Polich, 2007; Glazer et al., 2018). It can measure the limits of resource allocation during working memory processes (Daffner et al., 2011). Although there were no statistically significant changes in the reaction time and accuracy during the 1-back task after the intervention, the results indicated that Tai Chi practice improved the cognitive resource allocation ability and retrieval ability in the initial stage of the 1-back task in the elderly.

This study utilized ERP-fNIRS data and revealed that Tai Chi intervention improved the ability of older adults to integrate cognitive resources through neural electrical signals (P3) in a global working memory task and increased oxygenated hemoglobin concentration in the R-DLPFC during a 2-back task. As a result, the participants demonstrated faster reaction times and higher accuracy rates in the behavioral task. In comparison to normal older adults, those with mild cognitive impairment exhibited decreased activation of the frontal lobe in the 2-back task, indicating that high-load working memory tasks emphasize HbO in R-DLPFC (Yeung et al., 2016). This study suggests that Tai Chi intervention can enhance neural activity, promote attention resource allocation in older adults, and increase R-DLPFC HbO during more challenging working memory tasks, thereby improving their working memory ability.

Apart from the ERP and fNIRS techniques, researchers have demonstrated that aerobic exercise can regulate memory-related hormones, promote the production of brain-derived neurotrophic factor (BDNF), alter membrane receptor expression and translocation, activate multiple pathways, and modify synaptic plasticity to enhance memory (Loprinzi, 2018). MRI studies have also indicated that exercise intervention activates the hippocampus, which is associated with memory, and stimulates hippocampal neurogenesis (Friedl-Werner et al., 2020; Yue et al., 2020). Therefore, it is necessary to employ additional methods to investigate the effects of Tai Chi exercise on the working memory of older adults. Furthermore, future studies could consider including a young control group to provide a better understanding of age-related cognitive decline in older adults.

## 5. Conclusion

This study aimed to investigate the effects of Tai Chi exercise on working memory in elderly individuals by utilizing ERPs and fNIRS to detect underlying neural processes. Our findings suggest that after Tai Chi intervention, elderly individuals perform better in the 2-back working memory task. The neuroimaging results indicate that Tai Chi intervention can enhance neural activity and increase HbO in the R-DLPFC, as demonstrated by higher P3 amplitudes and activation in the R-DLPFC during the 2-back task. Tai Chi, a simple and low-risk mind-body exercise, can effectively alleviate cognitive decline in the elderly. However, further research is needed to understand the underlying mechanisms of these effects.

## Data availability statement

The raw data supporting the conclusions of this article will be made available by the authors, without undue reservation.

## Ethics statement

The studies involving human participants were reviewed and approved by the Ethics Committee of Capital University of Physical Education and Sports. The patients/participants provided their written informed consent to participate in this study.

## Author contributions

CJ, CW, and YY designed the experiment. CW, YY, and YD involved in data collection. CW, JM, XY, and YD completed the data analysis. CW and YD produced the first draft. CJ and XZ checked the manuscript and gave advice. All authors read and approved the final manuscript.
